# Supplementary material for: The genome sequence of the Large Wall Brown, Lasiommata maera (Linnaeus, 1758) (Lepidoptera: Nymphalidae)
Source: Wellcome Open Res. 2025 Aug 28;10:465. [Version 1] doi: 10.12688/wellcomeopenres.24746.1 (PMC12504934; doi:10.12688/wellcomeopenres.24746.1)
Supplement: Peer review history

# Reviewer report: Reviewer response for version 1

**DOI:** 10.21956/wellcomeopenres.27263.r132908

**Published:** 2025-10-27

**Authors:** Terrence Sylvester

**Affiliations:** 1 The University of Memphis, Memphis, Tennessee, USA

The authors present the genome sequence of the Large Wall Brown, *Lasiommata maera*. The background section provides a concise yet sufficient overview of the species’ distribution, morphology, feeding patterns, and developmental differences—key details that enhance understanding of the species’ basic biology and ecological context.

 The methods are clearly and effectively described. The authors follow a standard and logical workflow, beginning with genome property estimation from raw reads and proceeding through assembly and assessment. The sequencing depth appears adequate and supports the generation of a high-quality genome assembly.

 The assembly itself demonstrates excellent quality, as confirmed by standard assessment metrics. One point that could be clarified is the identification of the sex chromosome, specifically, the type of signal or analytical evidence used (whether a contact pattern unique to Z and W chromosomes, homology, read depth, or a combination of them), or a citation to a relevant methodological reference.

 Overall, this is a strong manuscript, and the genome represents a valuable resource for studies on genome evolution in Lepidoptera and insects more broadly.

Are sufficient details of methods and materials provided to allow replication by others?

Yes

Is the rationale for creating the dataset(s) clearly described?

Yes

Are the datasets clearly presented in a useable and accessible format?

Yes

Are the protocols appropriate and is the work technically sound?

Yes

Reviewer Expertise:

Genomics

I confirm that I have read this submission and believe that I have an appropriate level of expertise to confirm that it is of an acceptable scientific standard.

# Reviewer report: Reviewer response for version 1

**DOI:** 10.21956/wellcomeopenres.27263.r132909

**Published:** 2025-10-13

**Authors:** Arun Arumugaperumal

**Affiliations:** 1 Department of Biotechnology, Rajalakshmi Engineering College, Thandalam, Chennai 602105, Tamil NaduIndia, India

The authors have sequenced the whole genome of *Lasiommata maera* also known as the Large Wall Brown. The assembly reported here is of size 480.89 Mb. The specimen was a female and the W,Z sex chromosomes were also reported among the 29 chromosomes. The mitochondria genome was also reported of size 15.77 kb. The genome size is comparable to that of already reported genome of a *Lasiommata petropolitana* which had 492.84 Mb and 30 chromosomes[1]. The sample used for sequencing was clearly documented in figure 1. The authors have used long read Pac Bio sequencing and Hi-C sequencing to assemble the high quality genome. It is evident from the high contig N50 value.

 The following correction should me made. In Table 4, the EBP metric for haplotype 1 was mentioned as 6.7.Q64. But in the text EBP metric for haplotype 1 was mentioned as 6.C.Q64. Kindly correct it.

Are sufficient details of methods and materials provided to allow replication by others?

Yes

Is the rationale for creating the dataset(s) clearly described?

Yes

Are the datasets clearly presented in a useable and accessible format?

Yes

Are the protocols appropriate and is the work technically sound?

Yes

Reviewer Expertise:

Genomics; Bioinformatics

I confirm that I have read this submission and believe that I have an appropriate level of expertise to confirm that it is of an acceptable scientific standard, however I have significant reservations, as outlined above.

## References

[1] : The genome sequence of the Northern Wall Brown, Lasiommata petropolitana (Fabricius, 1787) (Lepidoptera: Nymphalidae). *Wellcome Open Research* .2025;10: 10.12688/wellcomeopenres.24734.1 10.12688/wellcomeopenres.24734.1 41477625 PMC12749566

# Reviewer report: Reviewer response for version 1

**DOI:** 10.21956/wellcomeopenres.27263.r132912

**Published:** 2025-10-07

**Authors:** Annabel Whibley

**Affiliations:** 1 The University of Auckland, Auckland, New Zealand; 2 Grapevine Improvement, Bragato Research Institute, Lincoln, New Zealand

In this Data Note, Escuer and colleagues present a high-quality reference genome assembly of a female individual of the Nymphalidae butterfly species *Lasiommata maera *(Large Wall Brown).

 The data collection, bioinformatics pipelines are report follow DToL/Project Psyche templating and are clear and comprehensive in their reporting of protocols and metadata. The Merian block visualisation also supports the structural integrity of the assembly. The standard combination of HiFi data (Revio) and HiC data is used as assembly input. Despite a high heterozygosity (2%) the assembly quality metrics are excellent, with haplotype 1 assembled to chromosome level and overall very high accuracy and completeness. No annotation has been released.  Public links are active.

 In the background section there is a typo: “The larva feeds on grasses as …” should be “The larva feeds on grasses such as …”

 I believe there is also a typo on the EBP summary value of Table 4. 6.7.Q64 should be 6.C.Q64.  I suspect this is a templated error since I have previously noted the same error in a different report.

Are sufficient details of methods and materials provided to allow replication by others?

Yes

Is the rationale for creating the dataset(s) clearly described?

Yes

Are the datasets clearly presented in a useable and accessible format?

Yes

Are the protocols appropriate and is the work technically sound?

Yes

Reviewer Expertise:

Genomics, Bioinformatics

I confirm that I have read this submission and believe that I have an appropriate level of expertise to confirm that it is of an acceptable scientific standard.

# Reviewer report: Reviewer response for version 1

**DOI:** 10.21956/wellcomeopenres.27263.r132917

**Published:** 2025-09-23

**Authors:** Marko Mutanen

**Affiliations:** 1 University of Oulu, Oulu, Finland

The genome report starts with an informative and nice species introduction which would serve as a good model for other similar works. I like that it introduces the species a bit more broadly than most genome reports.

 The genome quality is good. There is relatively high levels of heterozygosity. It would be interesting to know if this tells something about the patterns of taxonomic variability discussed in the Introduction, and if this possibly has resulted from admixture of two diverged populations. The Hi-C contact map show curious pattern of connection between W chromosome and chromosome 26 which apparently remains unexplained. The BUSCO percentage and other metrics suggest that the genome assembly is done well. The species could serve a nice model to focus on its population structure in more detail.

Are sufficient details of methods and materials provided to allow replication by others?

Yes

Is the rationale for creating the dataset(s) clearly described?

Yes

Are the datasets clearly presented in a useable and accessible format?

Yes

Are the protocols appropriate and is the work technically sound?

Yes

Reviewer Expertise:

Molecular taxonomy, DNA barcoding, species delimitation, dark taxa, phylogenetics, phylogenomics

I confirm that I have read this submission and believe that I have an appropriate level of expertise to confirm that it is of an acceptable scientific standard.
